# Protein Disulfide-Isomerase A3 Is a Robust Prognostic Biomarker for Cancers and Predicts the Immunotherapy Response Effectively

**DOI:** 10.3389/fimmu.2022.837512

**Published:** 2022-03-25

**Authors:** Zewei Tu, Qin Ouyang, Xiaoyan Long, Lei Wu, Jingying Li, Xingen Zhu, Kai Huang

**Affiliations:** ^1^ Department of Neurosurgery, The Second Affiliated Hospital of Nanchang University, Nanchang, China; ^2^ Jiangxi Key Laboratory of Neurological Tumors and Cerebrovascular Diseases, Nanchang, China; ^3^ Institute of Neuroscience, Nanchang University, Nanchang, China; ^4^ JXHC Key Laboratory of Neurological Medicine, Nanchang, China; ^5^ The Second Clinical Medical College of Nanchang University, Nanchang, China; ^6^ East China Institute of Digital Medical Engineering, Shangrao, China; ^7^ Department of Comprehensive Intensive Care Unit, The Second Affiliated Hospital of Nanchang University, Nanchang, China

**Keywords:** protein disulfide-isomerase A3 (PDIA3), pan-cancer, prognostic biomarker, immunotherapy response, CMap.

## Abstract

**Background:**

Protein disulfide isomerase A3 (PDIA3) is a member of the protein disulfide isomerase (PDI) family that participates in protein folding through its protein disulfide isomerase function. It has been reported to regulate the progression of several cancers, but its function in cancer immunotherapy is unknown.

**Methods:**

The RNA-seq data of cancer and normal tissues were downloaded from The Cancer Genome Atlas (TCGA) and the Genotype-Tissue Expression (GTEx) databases. The Cbioportal dataset was used to explore the genomic alteration information of PDIA3 in pan-cancer. Human Protein Atlas (HPA) and ComPPI websites were employed to mine the protein information of PDIA3, and western blot assay was performed to monitor the upregulated PDIA3 expression in clinical GBM samples. The univariate Cox regression and the Kaplan–Meier method were utilized to appraise the prognostic role of PDIA3 in pan-cancer. Gene Set Enrichment Analysis (GSEA) was applied to search the associated cancer hallmarks with PDIA3 expression. TIMER2.0 was the main platform to investigate the immune cell infiltrations related to PDIA3 in pan-cancer. The associations between PDIA3 and immunotherapy biomarkers were performed by Spearman correlation analysis. The immunoblot was used to quantify the PDIA3 expression levels, and the proliferative and invasive ability of glioma cells was determined by colony formation and transwell assays.

**Findings:**

PDIA3 is overexpressed in most cancer types and exhibits prognosis predictive ability in various cancers, and it is especially expressed in the malignant cells and monocytes/macrophages. In addition, PDIA3 is significantly correlated with immune-activated hallmarks, cancer immune cell infiltrations, and immunoregulators, and the most interesting finding is that PDIA3 could significantly predict anti-PDL1 therapy response. Besides, specific inhibitors that correlated with PDIA3 expression in different cancer types were also screened by using Connectivity Map (CMap). Finally, knockdown of PDIA3 significantly weakened the proliferative and invasive ability of glioma cells.

**Interpretation:**

The results revealed that PDIA3 acts as a robust tumor biomarker. Its function in protein disulfide linkage regulation could influence protein synthesis, degradation, and secretion, and then shapes the tumor microenvironment, which might be further applied to develop novel anticancer inhibitors.

## Introduction

According to the American Cancer Society, cancer death rate had fallen continuously over the past twenty years due to the advances in cancer treatment (1), but mounting cancer patients died every year. Blockade of immune checkpoints, such as cytotoxic T-lymphocyte associated protein 4 (CTLA-4) and programmed cell death protein-1 (PD-1) (2, 3), has made a great contribution in the immunotherapy of various cancers in recent years. However, a considerable number of cancer patients showed resistance to the currently available antigens and unable to achieve durable responses (4). Thus, exploring a novel immunotherapy biomarker or new immunoregulator genes can help cancer patients develop precise immunotherapy scheme and achieve more durable immunotherapy response.

Protein disulfide-isomerase (PDI) protein family is essential to the synthesis, folding, and degradation of proteins (5) and acts as oxidoreductase and molecular chaperone in the endoplasmic reticulum (6). It catalyzes the oxidation (formation) of disulfide bonds (7, 8), enabling it to mediate the folding of oxidative protein (9). Due to the critical role of PDI in protein folding, it has been demonstrated to be a prospective target for cancer therapy (10). The increased expression of PDI in many types of cancer enables PDI inhibition to repress the rapid growth of cancer cells, making PDI proteins become potential drug targets for the treatment of cancers (11, 12). PDI inhibition has been proved to be effective in the treatment of some cancers, such as glioblastoma (13, 14), neuroblastoma (15), and multiple myeloma (16).

Protein disulfide-isomerase A3 (PDIA3) (also known as ERp57, ERp60, and GRP58) is a member of the protein disulfide isomerase (PDI) family (17). The close association between PDIA3 and the occurrence and development of cancer has been corroborated by numerous studies. Higher expression of PDIA3 associates with poor survival outcomes in patients with multiple cancers, such as in diffuse gliomas (18), clear cell renal cell carcinoma (19), and hepatocellular carcinoma (HCC) (20). Overexpression of PDIA3 in GBM reduces the overall survival of patients with glioma by mediating macrophage/microglia pro-tumor activation (21). PDIA3 promotes the cell proliferation and inhibits apoptosis through the STAT3 signaling pathway to promote the progression of HCC (22). Expression of PDIA3 is strongly associated with cytotoxic T-lymphocyte dysfunction, and knockout of PDIA3 in the T cell can significantly enhance the antitumor activity in glioblastoma (23). Downregulation of PDIA3 inhibits proliferation and promotes apoptosis, making it a novel therapeutic target for colorectal cancer (CRC) and acute myeloid leukemia (AML) (24, 25). A recent study has demonstrated a positive correlation between PDIA3 and estimated scores, different stromal cell types, and invasive immunity in the cancer microenvironment in human gliomas (26). In conclusion, PDIA3 is one of the most reported PDI members in cancer progression and associated with immune evasion by T-cell killing (27). But the role of PDIA3 in cancer immune infiltrations and immunotherapy response prediction is not clear, and no comprehensive pan-cancer study has been conducted yet. We appraised the aberrant expression levels of PDIA3 in pan-cancer-compared human normal tissues and further examined the upregulated PDIA3 protein expression in clinical GBM samples. Besides, we also performed genomic alteration analysis, prognosis analysis, Gene Set Enrichment Analysis (GSEA), immune cell infiltration analysis, and potential drug sensitivity analysis of PDIA3 in pan-cancer; the most interesting finding is that PDIA3 can predict the immunotherapy response and might be a promising immunotherapy biomarker. Our results confirmed that PDIA3 is a robust prognostic biomarker for pan-cancer and predicts the immunotherapy response effectively, and it provided a main thread for further investigation on the role of PDIA3 in cancer immunity.

## Methods

### Data Source

The mRNA expression and clinical data of patients or samples from the TCGA pan-cancer cohort and Genotype-Tissue Expression (GTEx) datasets were downloaded from the UCSC Xena database (https://xenabrowser.net/datapages/). The 21 cancer cell lines’ transcriptomic profile is downloaded from the Cancer Cell Line Encyclopedia (CCLE) website (https://sites.broadinstitute.org/ccle/). To analyze the genomic alteration frequency of PDIA3 in the 33 cancer types, the web tool of cBioPortal for Cancer Genomics (http://cbioportal.org) was used. The Human Protein Atlas (HPA: https://www.proteinatlas.org/) database was used to affirm the distribution of PDIA3 protein at the subcellular level. The PDIA3 protein interaction information was obtained from the compartmentalized protein–protein interaction database (ComPPI) (http://comppi.linkgroup.hu). The abbreviations of cancers are represented in **Supplementary Table 1**. The associated R programming codes used in this manuscript were uploaded in the repository of GitHub (https://github.com/tzw2019/Pancancer-PDIA3).

### GBM Sample Collection

A total of seven GBM samples with adjacent tissues were resected from inpatients who were under treatment in the Neurosurgery Department of The Second Affiliated Hospital of Nanchang University (NCUSAH) in 2021. The tumor excisions were stored in liquid nitrogen after excising from GBM patients. Informed consents were acquired from inpatients enrolled in this study. The usage of clinical excisions was consented by the Medical Ethics Committee of NCUSAH. The processes of clinical sample collection and usage were in strict accordance with the guideline.

### Single-Cell Analysis of PDIA3

Related single-cell analysis was applied by the Tumor Immune Single-cell Hub (TISCH) web tool (28). The analysis parameters were as follows: PDIA3 (Gene), major lineage (Cell-type annotation), and all cancers (Cancer type). The expression levels of PDIA3 in each cell type were quantified and visualized by a heatmap, scatter diagrams, and violin plots. The data collection, processing, and cell annotation procedures were introduced in the document part of the TISCH website (http://tisch.comp-genomics.org/documentation/).

### Prognosis Analysis of PDIA3 in Pan-Cancer

Four types of prognosis data including overall survival (OS), disease-specific survival (DSS), disease-free interval (DFI), and progression-free interval (DFI) were curated from the UCSC Xena database (https://xenabrowser.net/datapages/) information. Then univariate Cox regression and the Kaplan–Meier model were used to assess the prognostic role of PDIA3 for a specific prognosis type in each cancer. The continuous variable of PDIA3 expression data was used in the univariate Cox regression. Moreover, bivariate PDIA3 expression levels were used to perform Kaplan–Meier curve analysis, whose cutoff was chosen by the “surv-cutpoint” function of the “survminer” R package (0.4.9). The log-rank p value of the K-M method and the hazard ratio (HR) with 95% confidence interval (95%CI) was computed, and the outcomes were presented as a heatmap.

### Identification of Differential Expression Genes Between Low- and High-PDIA3 Subgroup

To identify genes differentially expressed between low- and high-PDIA3 subgroups in each cancer, cancer patients were ordered by PDIA3 mRNA expression, and the top 30% patients were defined as the high-PDIA3 subgroup and the bottom 30% as the low-PDIA3 subgroup. The log2(fold change) and the adjusted p-value of each gene were obtained in each cancer type by performing differential expression analysis using the “limma” R package. Genes with p-adjusted values < 0.05 were regarded as differential expression genes (DEGs). The DEGs between low- and high-PDIA3 subgroups in each cancer are concluded in **Supplementary Table 2**.

### Gene Set Enrichment Analysis

The “gmt” file of the hallmark gene set (h.all.v7.4.symbols.gmt), which contains 50 hallmark gene sets, was downloaded from the website of Molecular Signatures Database (MSigDB, https://www.gsea-msigdb.org/gsea/index.jsp) and used to calculate the normalized enrichment score (NES) and false discovery rate (FDR) of the DEGs between low- and high-PDIA3 expression cancer groups for each biological process in each cancer type. The GSEA was conducted using the R package “clusterProfiler” (29), and the results were summarized in the bubble plot depicted by the R package “ggplot2”.

### Immune Cell Infiltration Analysis in TIMER2

Tumor IMmune Estimation Resource (TIMER) is a data source for quantifying immune cell infiltrations across distinct cancers. The immune cell infiltration levels of TCGA cancers were downloaded from the TIMER2 database (http://timer.cistrome.org/). We analyzed the correlations between PDIA3 mRNA expression and 21 immune cell subsets including CD4+ T cells, cancer-associated fibroblast (CAF), progenitors of lymphoid, progenitors of myeloid, progenitors of monocyte, endothelial cells (Endo), eosinophil (Eos), hematopoietic stem cells (HSC), T-cell follicular helper, γ/δ T cells, NK T cells, regulatory T cells (Tregs), B cells, neutrophils, monocytes, macrophages, dendritic cells, NK cells, Mast cells, and CD8+ T cells in pan-cancer by Spearman correlation analysis.

### Immunotherapy Prediction Analysis

The Spearman correlation analysis was utilized to calculate the statistical correlations between PDIA3 and well-known immunotherapy biomarkers, like tumor mutation burden (TMB), microsatellite instability (MSI), and other well-known immune checkpoint genes, in pan-cancer. Two immune checkpoint blockade (ICB) therapy cohorts were obtained to validate the immunotherapy response prediction ability of PDIA3. The IMvigor210 cohort contains 288 urological cancer patients treated with atezolizumab (anti-PDL1), and the GSE91061 cohort includes 51 melanoma patients’ transcriptomic profile before receiving nivolumab (anti-PD1).

### Compounds Correlating With PDIA3 in Pan-Cancer

The Connectivity Map (CMap), a new tool for biomedical research, can be employed to connect diseases with the effective drugs (30). In this study, CMap (https://portals.broadinstitute.org/cmap/) was used to identify the relationships between PDIA3 expression levels and specific inhibitors in pan-cancer. The detailed methods and processes to apply the web tool and visualize the heatmap were provided in a previous study (31).

### Cell Culture, Plasmids, and Western Blot

The LN229 cell line was bought from the Chinese Academia Sinica Cell Repository (Shanghai, China), and the SW1088 cell line was purchased from the American Type Culture Collection (ATCC). The cells were cultured as described in our previous studies (32, 33). The PDIA3 knockdown plasmids were designed and purchased from the Sheweisi Biotechnology Company (Tianjin, China). The rabbit polyclonal anti-PDIA3 (15967-1-AP, Proteintech, China) and rabbit anti-GAPDH (1:5,000, 10494-1-AP, Proteintech) were purchased and used for western blot assay in a diluent concentration of 1:2,000 and 1:5,000, respectively. The immunoblot assay was completely consistent with the workflow described in a previous study we conducted (32).

### Colony Formation and Transwell Assays

For colony formation assays, clonogenic growth was determined by plating 200 cells in 5 ml of a complete medium in 6-well cell culture dishes. We replaced the culture medium twice a week, cultured for around two weeks. A cluster with cells of more than 40 is defined as a clonogenic colony cell cluster. Then the colony cell clusters were imaged, and colony numbers were calculated by the ImageJ software.

Twenty-four-well transwell inserts with an 8-mm pore size (Corning, USA) were used to perform Transwell Matrigel invasion assays. A 24-well permeable support plate was coated with 200 mg/ml Corning Matrigel matrix (Corning, USA) followed by incubation at 37°C for 5 h to solidify the Matrigel matrix. Glioma cells (1 × 10^5^) in a serum-free medium were added to the transwell upper chamber, with the lower chamber containing a medium with 10% FBS. After incubation for 6 h at 37°C, the migrated cells present on the underside of the transwell membrane were stained with crystal violet, imaged, and counted using ImageJ.

### Statistical Analysis

To compare the expression levels of PDIA3 between normal tissues and tumor tissues, Wilcoxon rank-sum test was performed to calculate statistical significance. Paired t-test was used to evaluate the statistical significance between PDIA3 protein expression levels of clinical GBM samples and adjacent tissues. Univariate Cox regression analysis and the Kaplan–Meier method were employed to assess the prognostic of PDIA3 expression in each cancer. Spearman correlation analysis was performed to assess the statistical relationships between PDIA3 and other factors. To compare the proportions of an ICI-therapy responder and a nonresponder between low- and high-PDIA3 cancer subgroups, chi-square test was used to compute the statistical significance.

## Results

### Basic Information of PDIA3

**Figure 1:**
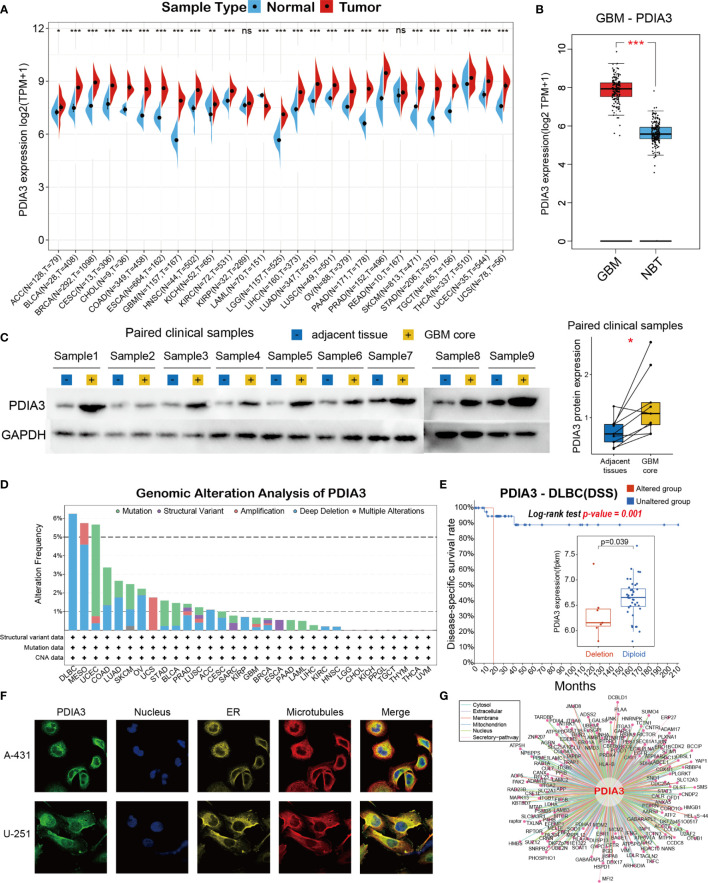
Basic information of PDIA3. **(A)** The expression level of PDIA3 between tumor and normal tissues in each cancer based on the integrated data from TCGA and GTEx datasets. **(B)** The expression level of PDIA3 between GBM and normal brain tissues. **(C)** Western blot protein detection of the PDIA3 expression levels in paired GBM and adjacent normal tissues. **(D)** PDIA3 alteration frequency analysis in pan-cancer study according to the cBioPortal database. **(E)** The PDIA3 expression levels and prognosis of DSS between PDIA3-deletion and diploid DLBC patients. **(F)** The immunofluorescence images of PDIA3 protein, nucleus, endoplasmic reticulum (ER), microtubules and the merged images in A-431 and U251 cell lines. **(G)** The protein-protein interaction (PPI) network presents the proteins interacting with PDIA3. The labelled asterisk indicated the statistical p value (ns p > 0.05, *p < 0.05, **p < 0.01 and ***p < 0.001). ns, nonsense.

To firstly comprehend the basic information of PDIA3 in cancer, we employed the TCGA and GTEx databases to evaluate the expression level of PDIA3 in cancers compared with normal tissues. High expression levels of PDIA3 were examined in the majority of TCGA cancers, including ACC, BLCA, BRCA, CESC, CHOL, COAD, ESCA, GBM, HNSC, KIRC, LGG, LIHC, LUAD, LUSC, OV, PAAD, PRAD, SKCM, STAD, TGCT, THCA, UCEC, and UCS. By contrast, low PDIA3 expression was observed only in LAML (**Figure 1A**). Compared with normal tissues, the mRNA expression of PDIA3 in GBM is most significantly upregulated (**Figure 1B**) across the 27 types of cancers, so we further performed western blot assay in clinical GBM samples to confirm the protein expression of PDIA3 in GBM samples compared with adjacent tissues (**Figure 1C**), and the result indicated that the PDIA3 protein was upregulated in GBM samples as expected. To investigate the expression levels of PDIA3 among different tissues and cancer cell lines, we compared the PDIA3 expression levels among 31 human normal tissues and 21 cancer cell lines using the data downloaded from the GTEx and CCLE datasets.

Then genomic alteration analysis of PDIA3 indicated that the alterations of PDIA3 across pan-cancer were not universal, and the most frequently altered cancer type was lymphoid neoplasm diffuse large B-cell lymphoma (DLBC), exceeding 6% DLBC patients and was mostly deep deletion (**Figure 1D**). To analyze the impacts of PDIA3 alterations in the expression of PDIA3 and the prognosis of DLBC patients, we further analyzed the PDIA3 expression levels and DSS time between PDIA3-deletion and diploid DLBC patients. Our results suggested that the PDIA3 expression was downregulated in PDIA3-deletion samples, and DSS time and rate of PDIA3-deletion DLBC patients were significantly lower than those of normal diploid DLBC patients (**Figure 1E**, p=0.01, log-rank test). Furthermore, immunofluorescence (IF) images showed that PDIA3 protein was mainly localized and distributed in the endoplasmic reticulum (ER) in A-431 and U251 tumor cell lines (**Figure 1F**). Finally, a protein–protein interaction (PPI) network was conducted based on the interaction data obtained from the ComPPI website, describing that the subcellular localization of protein closely related to PDIA3 was distributed in cytosol, mitochondria, nucleus, extracellular, secretory pathway, and membrane (**Figure 1G**).

### Single-Cell Analysis of PDIA3 in Cancers

**Figure 2:**
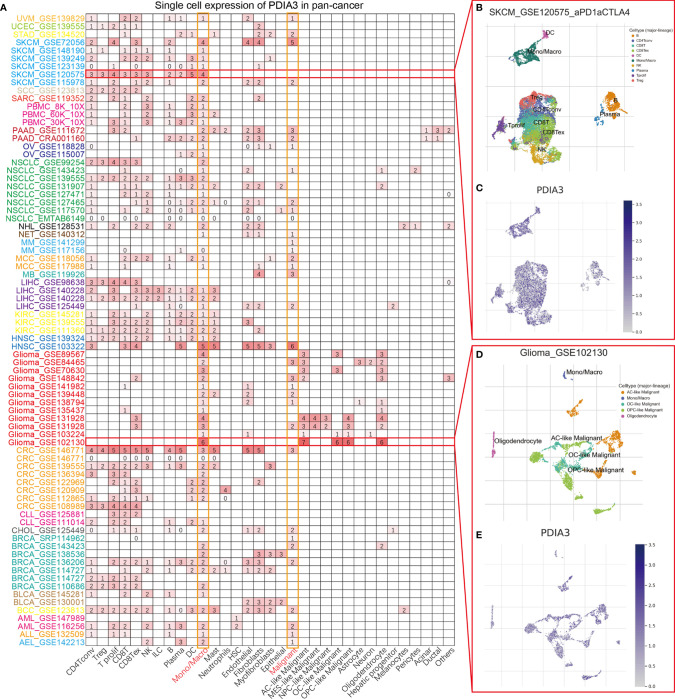
**(A)** Summary of PDIA3 expression of 33 cell types in 79 single cell datasets. **(B)** Scatter plot showed the distributions of 10 different cell types of the GSE120575 SKCM dataset. **(C)** Scatter plot showed the PDIA3 expression levels of cells in the GSE120575 dataset. **(D)** Scatter plot showed the distributions of 5 different cell types of the GSE102130 glioma dataset. **(E)** Scatter plot showed the PDIA3 expression levels of cells in the GSE120575 dataset.

To understand the main cell types that express the PDIA3 in cancer microenvironments, we performed the single-cell analysis of PDIA3 in 79 single-cell datasets of cancer samples. The heatmap depicted in **Figure 2A** represents the expression levels of PDIA3 of 33 cell types (including immune cells, stromal cells, malignant cells, and functional cells) in 79 datasets using the TISCH web tool. The results indicated that PDIA3 was mainly expressed in the immune cells (especially monocyte/macrophage) and malignant cells. Pointedly, in the GSE120575 dataset, which contains 16,291 cells from 32 metastatic skin cutaneous melanoma (SKCM) patients treated with immune checkpoint inhibitors, PDIA3 expression is widely expressed across immune cell types like T cells, dendritic cells NK cells, monocytes, or macrophages in the SKCM microenvironment (**Figures 2B, C**). In the GSE102130 glioma dataset, we analyzed 3,321 cells from 6 glioma patients; PDIA3 is highly expressed in malignant cells and monocytes/macrophages in the glioma microenvironment (**Figures 2D, E**).

### Prognostic Analysis of PDAI3 in Pan-Cancer

**Figure 3:**
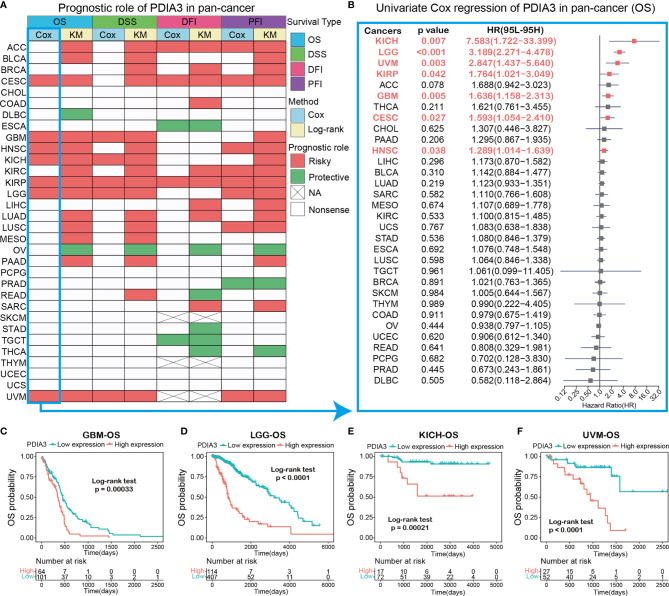
**(A)** Summary of the correlation between expression of PDIA3 with overall survival (OS), disease-specific survival (DSS), disease-free interval (DFI) and progression-free interval (PFI) based on the univariate Cox regression and Kaplan-Meier models. Red indicates that PDIA3 is a risk factor affecting the prognosis of cancer patients, and green represents a protective factor. Only p values < 0.05 are shown. **(B)** The forest plot exhibited the prognostic role of PDIA3 in cancers by univariate Cox regression method. The cancer type in red represents the PDIA3 acts as a risky factor with statistical significance. **(C–F)** Kaplan-Meier overall survival curves of PDIA3 in GBM **(C)**, LGG **(D)**, KICH **(E)** and UVM **(F)**.

The heatmap showing the prognosis analysis of PDIA3 in pan-cancer indicated that PDIA3 is highly associated with the prognosis of most cancers except CHOL, PCPG, SKCM, THYM, UCEC, and UCS (**Figure 3A**). The results of OS analysis suggested that PDIA3 is a perilous factor for patients with ACC, BLCA, CESC, GBM, HNSC, KICH, KIRC, KIRP, LGG, LUAD, LUSC, MESO, PAAD, and UVM while a protective factor for patients with DLBC and OV. Since the survival outcome endpoints of OS included many noncancer deaths, we then performed DSS analysis, which has a much greater relevance to the efficacy of cancer treatment. The results of DSS analysis were highly consistent with those of OS analysis; OS analysis suggested that PDIA3 is a risk factor for the prognosis of the above 14 cancers, and coincidentally, DSS analysis also indicated that PDIA3 is a risk factor for these cancers. DFI and PFI outcomes were also examined to fully demonstrate that PDIA3 is a risk factor in most cancer types and significantly related to the prognosis of cancer patients. Furthermore, the outcomes of OS, DSS, PFI, and DSS all showed that PDIA3 was a protective factor for ovarian cancer (OV). Studies have shown that the silence of PDI can cause cytotoxicity in ovarian cancer cells (34), illustrating the important role of PDIA3 in the prognosis of OV patients and its potential as a prognostic biomarker.

To gain further insight into how PDIA3 affects patient prognosis, univariate Cox regression was utilized to analyze the prognosis across 32 TCGA cancer types. The results shown in the forest plot suggested that the downregulation of the PDIA3 expression has remarkable relationships with OS time prolongation in CESC (HR = 1.593[95%CI, 1.054 to 2.410], p = 0.027), GBM (HR = 1.636[95%CI, 1.158 to 2.313], p = 0.005), HNSC (HR = 1.289[95%CI, 1.014 to 1.639], p = 0.038), KICH (HR = 7.583[95%CI, 1.722 to 33.399], p = 0.007), KIRP (HR = 1.764[95%CI, 1.021 to 3.049], p = 0.042), LGG (HR = 3.189[95%CI, 2.271 to 4.478], p < 0.001), and UVM (HR = 2.847[95%CI, 1.437 to 5.640], p = 0.003) (**Figure 3B**). Several studies indicate that PDIA3 is closely related to the progression and prognosis of gliomas (21, 23), so we further performed Kaplan–Meier curve analysis of GBM and LGG, which suggested that a higher PDIA3 expression was associated with poor survival outcomes (**Figures 3C, D**), indicating that PDIA3 is a prognostic biomarker of OS outcomes in LGG and GBM. Furthermore, Kaplan–Meier survival analysis also validated that the higher PDIA3 expression in KICH (**Figure 3E**) and UVM (**Figure 3F**) correlated with poor OS prognosis.

### GSEA of PDIA3 in Pan-Cancer

**Figure 4:**
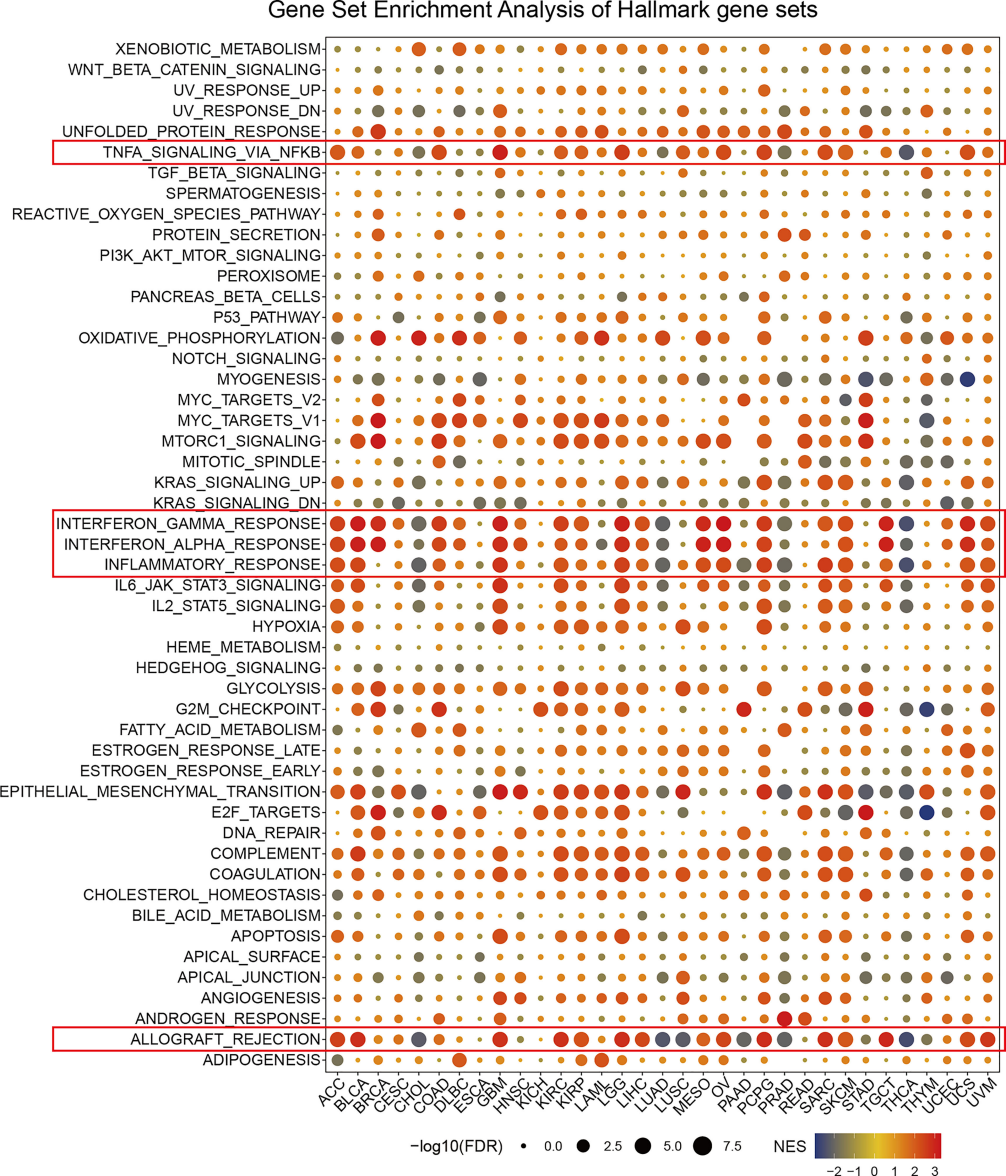
The hallmarks gene set enrichment analysis of PDIA3 in pan-cancer. The size of circle represents the FDR value of enrich term in each cancer, and the color indicates the normalized enrichment score (NES) of each term.

The differential expression genes (DEGs) between low- and high-PDIA3 subgroups in each cancer were used to perform GSEA in order to discern the PDIA3-associated cancer hallmarks. We found that PAID3 expression was remarkably related to immune-related pathways, such as TNFA-signaling-via-NFKB, IFN-α response, IFN-γ response, inflammatory-response, and allograft-rejection pathways, especially in ACC, BLCA, GMB, LGG, SKCM, and UCS (**Figure 4**). These data uncovered a potential association between PDIA3 expression and immune activation in the tumor microenvironment (TME). In addition, epithelial-mesenchymal transition (EMT) hallmark was significantly enriched in high-PAID3 subgroups of ACC, BLCA, CESC, GBM, HNSC, KIRC, KIRP, LAML, LGG, LUSC, PCPG, SARC, SKCM, THYM, and UVM. In previous studies, EMT has been significantly confirmed to be related to the occurrence, metastasis, and drug resistance of cancers (35, 36), suggesting that PDIA3 might play an indispensable part in the oncogenesis and development of cancers by enrolling in EMT. Besides, oxidative phosphorylation, unfolded protein response, MYC targets, and MTORC1 signaling were also tightly associated with PDIA3 expressions in cancers. In conclusion, these results indicated that a higher PDIA3 expression is associated with the immune-activation status of cancers and might provide some clues for further investigation of the functions and roles of PDIA3 in cancer initiation and progression.

### TIMER Immune Cell Infiltration Analyses

**Figure 5:**
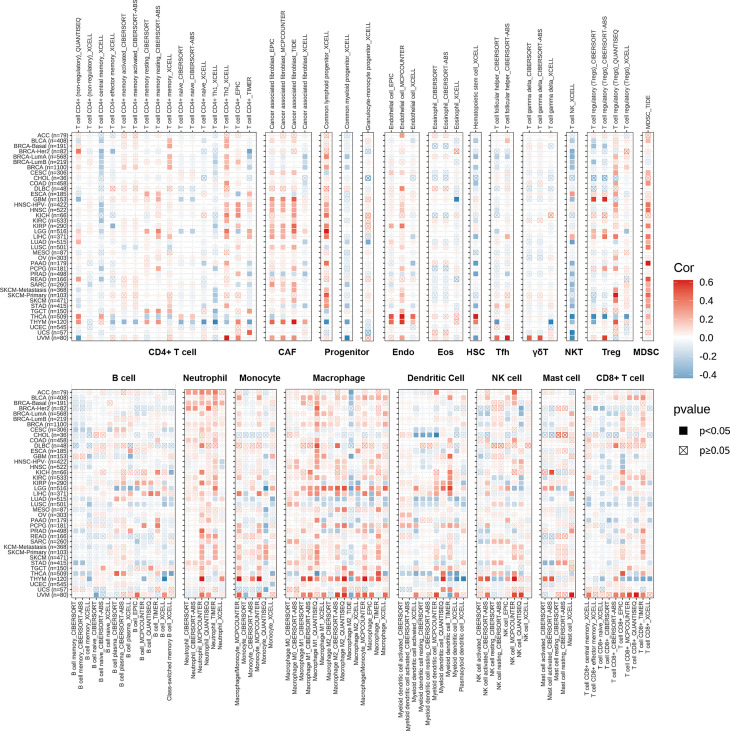
The correlations of PDIA3 expression and the infiltration levels of CD4+ T cells, CAF, progenitor, Endo, Eos, HSC, Tfh, γδT, NKT, regulatory T cells (Tregs), B cells, neutrophils, monocytes, macrophages, dendritic cells, NK cells, Mast cells and CD8+ T cells in cancers. Positive correlation in red and negative correlation in blue.

To illustrate the relationships between PDIA3 and cancer immunity, we further excavate the correlations between PDIA3 expression and immune cell infiltrations. Spearman correlation analyses were conducted utilizing pan-cancer immune cell infiltration data from the TIMER2 database. The outcomes revealed the infiltration levels of CD4+ T cells, CAF, progenitors of lymphoid, progenitors of myeloid, progenitors of monocyte, Endo, Eos, HSC, Tfh, γ/δT, NK T cells, Tregs, B cells, neutrophils, monocytes, macrophages, dendritic cells, NK cells, Mast cells, and CD8+ T cells in pan-cancer (**Figure 5**). The results indicated that PDIA3 was positively associated with the infiltration levels of CD4+ T cells, CAF, progenitors of lymphoid, MDSC, neutrophils, and macrophages in most of the TCGA cancers and negatively associated with the infiltration levels of NK T cells in most TCGA cancers especially in THCA, UCS, and UVM. Moreover, in THYM and UVM, PDIA3 was positively related to the infiltration level of most immune cells such as CAF, Endo, Eos, Tregs, neutrophils, monocytes, macrophages, dendritic cells, NK cells, and mast cells. In recent years, studies have indicated that CD4+T cells, CAF, MDSC, neutrophils, and macrophages play a key role in cancer immunotherapy (37–41); the role of immune cells in cancer therapy should not be overlooked. Our results indicate that PDIA3 might affect the development, prognosis, and therapy of cancers by associating with immune cells.

### Relationships Between PDIA3 and Immune Regulators, TMB, and MSI

**Figure 6:**
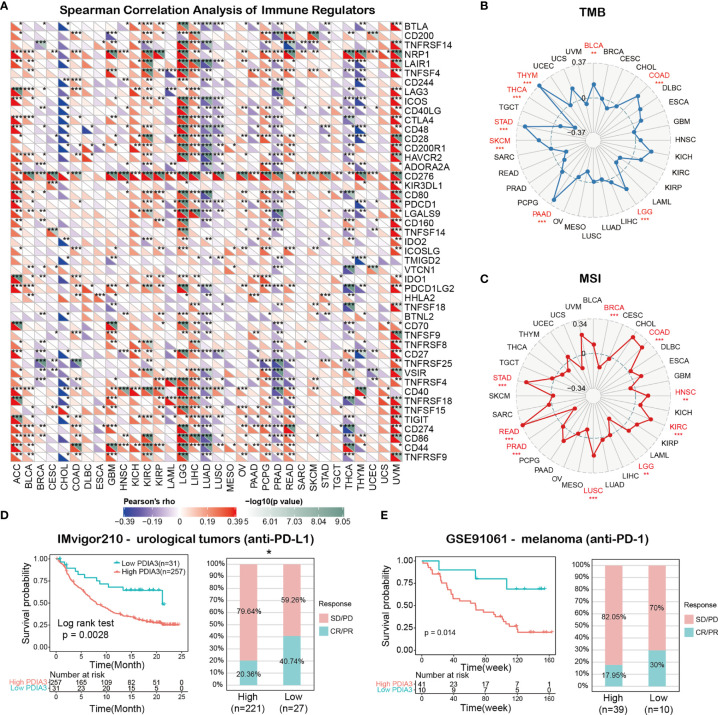
**(A)** The spearman correlation heatmap shows the correlations between the PDIA3 expressions and the 47 types of immune regulators in pan-cancer. Red represents positive correlation and blue represent negative correlation. **(B)** Correlations between PDIA3 expression and tumor mutation burden in pan-cancer. **(C)** Correlations between PDIA3 expression and microsatellite instability in pan-cancer. **(D)** Kaplan-Meier curves for low- and high-PDIA3 patient groups in IMvigor210 cohort (anti-PD-L1, urological), and the fraction of urological tumors patients with response to anti-PD-1 therapy in low- and high-PDIA3 subgroups of IMvigor210 cohort. **(E)** Kaplan-Meier curves for low- and high-PDIA3 patient groups in GSE91061(anti-PD-L1, melanoma), and the fraction of melanoma patients with response to anti-PD-1 therapy in low- and high-PDIA3 subgroups of GSE91061. The labelled asterisk indicated the statistical p value (*p < 0.05, **p < 0.01, ***p < 0.001).

The associations between PDIA3 and 47 immune regulators in pan-cancer are displayed in **Figure 6A**. We found that PDIA3 had a strong positive relationship with most immune regulators in ACC, LGG, and UVM and a strong negative relationship with most immune regulators in CHOL, LUAD, PRAD, THCA, and THYM. Besides, there was a robust positive relationship between PDIA3 and CD276 and Neuropilin-1(NRP1) in most of the TCGA cancers. To understand the role of PDIA3 in predicting the efficiency of immune checkpoint inhibitors (ICIs), we further assessed the correlation between PDIA3 expression and TMB and MIS. Positive correlations with TMB were identified in LGG, COAD, BLCA, THYM, THCA, STAD, SKCM, and PAAD (**Figure 6B**). Moreover, for the correlation between PDIA3 expression and MSI, positive associations were discovered in COAD, HNSC, KIRC, LUSC, READ, and STAD, and negative correlations were discovered in BRCA and LGG (**Figure 6C**). Our results suggested that PDIA3 has the potential to predict the efficiency of ICIs in the corresponding cancers.

### The Predictive Role of PDIA3 in Cancer Immunotherapy

Immune checkpoint inhibitors (ICIs), such as the anti-PD-L1 antibodies, have made a great contribution to the immunotherapy of cancers (42, 43). In view of the results above, we subsequently analyze the predictive role of PDIA3 in ICI cancer cohorts. The relationship between PDIA3 and anti-PDL1 therapy response in patients with urological tumors indicated that the survival rate and time of PDIA3 low-expression patients were better than those of high-expression patients (**Figure 6D**). In the urological tumors of the IMvigor210 cohort, the response rate to anti-PD-L1 therapy was 20.36% in high-PDIA3 expression patients, which is significantly lower than 40.74% in low-PDIA3 expression patients. Furthermore, similar results were found in melanoma patients treated with anti-PD-1 therapy. In the GSE91061 melanoma cohort (**Figure 6E**), melanoma patients with low-PDIA3 expression had much better survival probability than patients with high-PDIA3 expression patients, and the response rate to anti-PD-1 was 17.95% in the PDIA3 high-expression subgroup, while 30% patients responded to the anti-PD1 therapy in the PDIA3 low-expression subgroup. These data confirmed the potential ability of PDIA3 in immunotherapy response prediction and indicated that PDIA3 is a promising biomarker for cancer immunotherapy.

### Connectivity Map (CMap) Analysis of PDIA3 in Pan-Cancer

**Figure 7:**
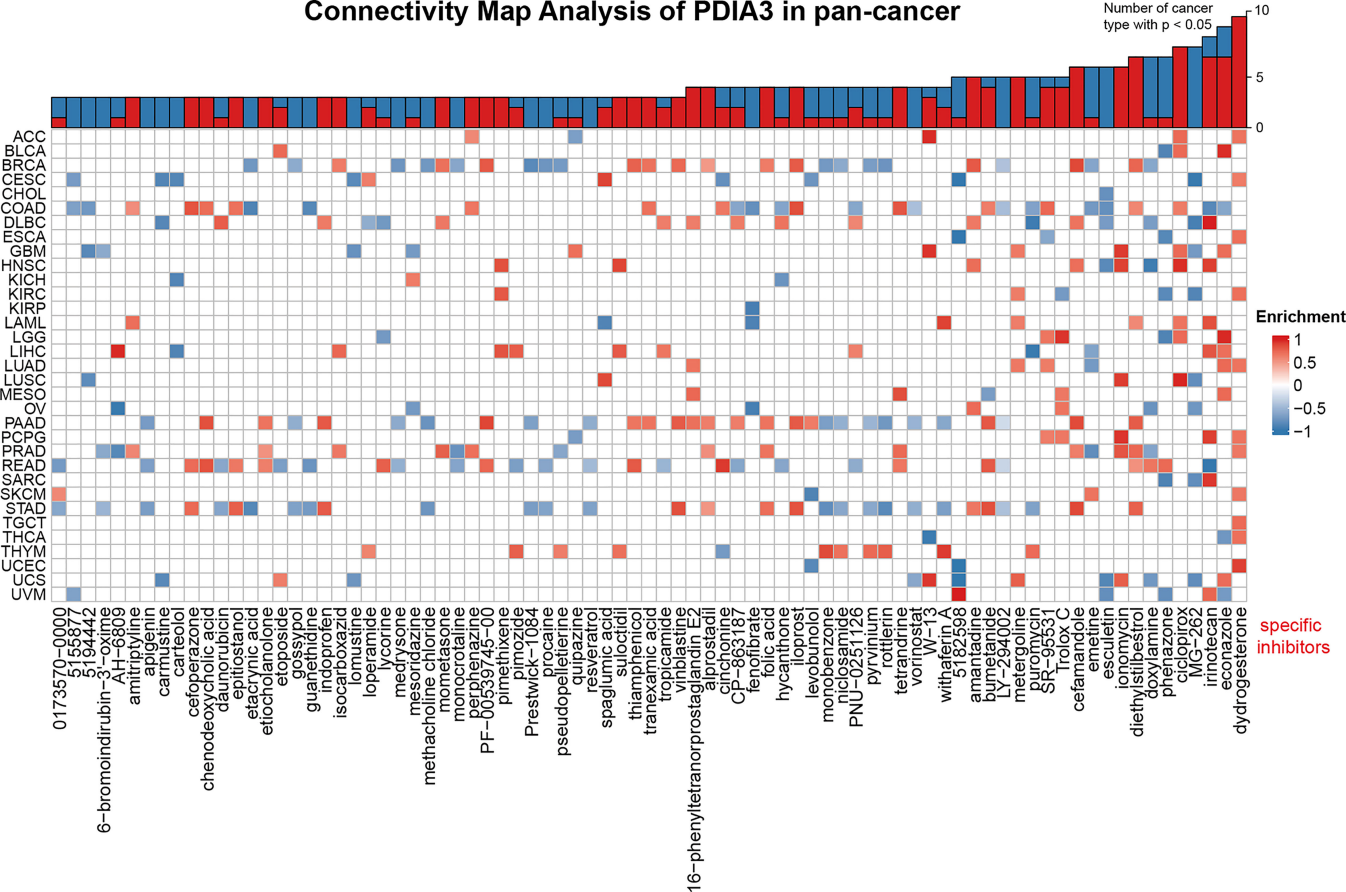
Heatmap representing enriched score (positive in blue, negative in red) of each drug from the CMap database for each cancer. Components or drugs are ordered by decreasing number of enriched cancers from right to left.

Potential components targeting PDIA3 in pan-cancer are presented as a heatmap in **Figure 7** by using the data downloaded from the CMap dataset. Drugs or components that might target to PDIA3 in more than three cancer types were represented in the heatmap, and detailed parameters of enriched components in each cancer are shown in **Supplementary Table 3**. Dydrogesterone is significantly enriched in 11 cancer types especially in UCEC, and ciclopirox is highly concentrated in 8 cancers especially in LUSC. In addition, diethylstilbestrol, econazole, irinotecan, ionomycin, and cefamamdole were enriched in at least 6 cancers. These drugs have been more or less used in the prevention and treatment of several cancers; for example, dydrogesterone is used to reduce the risk of breast cancer (44), and ciclopirox is utilized to inhibit the growth of human papillomavirus (HPV)-positive cancer cells (45). The associations between these components and PDIA3 should be paid with more attention. Our results indicate that the existing clinical application of these drugs is only the tip of the iceberg, and their roles in the oncogenesis and development of different cancers and potential targets as well as underlying mechanisms need to be further explored.

### PDIA3 Promotes Glioma Cell Proliferation and Invasion

To reveal the role of PDIA3 in glioma cell proliferation and invasion, we designed the following assays. Firstly, we used the western blot assay to identify the knockdown status of PDIA3 in the LN229 and SW1088 cell lines. **Supplementary Figure 2A** showed that sh-2 and sh-3 shRNA could significantly knock down the protein level of PDIA3 in glioma cell lines. To uncover the potential ability of PDIA3 in regulating glioma proliferation and invasion, we performed the colony formation and transwell assays to reflect the proliferative and invasive ability of glioma cells. Colony formation analysis indicated that the proliferative ability of LN229 and SW1088 cells both decreased after PDIA3 was knocked down (**Supplementary Figures 2B, C**). Besides, transwell assays also proved that knockdown of PDIA3 significantly weakened the invasive ability of LN229 and SW1088 glioma cell lines (**Supplementary Figures 2D, E**). Thus, we believed that PDIA3 is vital for glioma cell proliferation and invasion.

## Discussion

Immune checkpoint blockade and CAR-T cell therapy have made great breakthroughs in cancer immunotherapy (46), enabling cancer patients to see the hope of cure. However, immunotherapy only works for a subset of cancers, and only a small portion of cancer patients react well to immunotherapy due to the heterogeneity of the tumor suppression microenvironment in each patient. Biomarkers that employed to accurately forecast a patient’s response to immunotherapy will make individualized immunotherapy possible for patients. In this study, we discovered that PDIA3 is a robust prognostic biomarker for pan-cancer, and it can predict the immunotherapy response effectively. Moreover, our results could provide some clues for further investigation to recover the potential role of PDIA3 in cancer immunity and immunotherapy.

Firstly, we assessed the expression level of PDIA3 in pan-cancer based on TCGA and GTEx data. The outcomes revealed a clear increase of the PDIA3 expression in most cancer types, except KIRP, LAML, and READ. The upregulation of PDIA3 in GBM was particularly pronounced when compared to normal brain tissues. We also conducted a clinical sample test with GBM samples, and western blot analysis suggested that the expression of PDIA3 in nine GBM tissues was remarkably higher than that in normal tissues adjacent to cancer, further validating the high protein expression of PDIA3 in GBM. Low expression of PDIA3 can be observed in DLBC patients with PDIA3 deep deletion, which is associated with the poor DSS of DLBC patients. These results reveal that PDIA3 is aberrantly upregulated and expressed in most cancers, which might endow cancer cells with the ability to resist the endoplasmic reticulum stress (ERS), leading to cancer cell survival in a severe microenvironment.

Then, we assessed the association between PDIA3 and prognosis of cancer patients. Results from OS, DSS, DFI, and PFI analyses were highly consistent, showing that PDIA3 is significantly associated with the prognosis of cancer patients, and PDIA3 is a risk factor for a large proportion of cancers. Combined with the previous PDIA3 expression analysis, we can observe that a high expression of PDIA3 results in worse prognosis in ACC, BLCA, BRCA, CESC, COAD, GBM, HNSC, KIRC, LGG, LIHC, LUAD, LUSC, and PAAD. The Kaplan–Meier survival curves of LGG and GBM clearly demonstrated the correlation between high PDIA3 expression and poor prognosis. A previous study also has proved that 17 members of PDI have the potential to become biomarkers for the diagnosis of gliomas, including LGG and GBM (47). The above results suggested that PDIA3 plays an important part in predicting the prognosis of cancer patients and could become a robust prognostic biomarker for cancer patients.

The GSEA result suggests that PDIA3 is closely associated with immune-activated processes, such as TNFA-signaling-via-NFKB, IFN-α response, IFN-γ response, inflammatory-response, and allograft-rejection pathways, but completely opposite results were observed in distinct cancer types. For example, these processes were mostly significantly enriched in high-PDIA3 cancer subgroups, but reversed results were found in CHOL, LUAD, PRAD, PAAD, and THCA. This indicated that PDIA3 might play different roles in distinct cancer types. The research by Quan et al. also has demonstrated that immune-related pathways, such as IFN-α response, IFN-γ response, and TNFA signaling-via-NFKB pathway, played irreplaceable roles in the prediction of prognosis, immune infiltration, and immunotherapy reaction of LGG patients with epilepsy (48), which could support a part of our findings.

Another essential discovery of our study is that the expression of PDIA3 is highly related to immune infiltration in pan-cancer. PDIA3 is associated positively with the degree of infiltration of CD4+ T cells, CAF, MDSC, neutrophils, and macrophages in most cancers, indicating that PDIA3 is most likely to affect the development and prognosis of cancers by shaping the tumor microenvironment. Besides, the correlation analysis of PDIA3 and immune regulators in pan-cancer suggested that PDIA3 expression is correlated with many immune regulator gene expressions, especially in ACC, LGG, and UVM. In previous studies, targeting CD276 and Neuropilin-1(NRP1) has achieved remarkable success in tumor immunotherapy (49, 50); combined with our finding that PDIA3 is significant correlated with the expression of CD276 as well as NRP1 in many cancer types, we speculate that the potential association between PDIA3 and CD276 is promising for our further investigation.

We further analyzed the expression level of PDIA3 and response to anti-PD-L1 immunotherapy at urological tumors and anti-PD1 in melanomas. The results show that PDAI3 is a powerful prognostic biomarker in urological tumors and can effectively predict the response to anti-PD-L1 immunotherapy, and consistent results were discovered in melanoma patients treated with anti-PD-1. Our results showed that PDIA3 is a powerful biomarker to predict response to immune checkpoint blockage therapy. Therefore, we believe that PDIA3 could be a robust immunotherapy biomarker for cancers, and it possesses the strong potential to be applied in clinical cancer treatment.

Subsequently, we identified specific inhibitors to screen molecular targets that may eventually lead to novel anticancer inhibitors. The PDIA3-associated potential drug sensitivity analysis suggested that dydrogesterone, ciclopirox, and diethylstilbestrol have shown great potential in developing new molecular targets. It has been revealed in a previous study that dydrogesterone is an immunomodulator that promotes the production of Th2 and inhibits the production of Th1 and Th17 (51). Therefore, we hypothesize that dydrogesterone has an effect on the tumor microenvironment. In recent years, the antifungal drug ciclopirox olamine (CPX) has been repositioned as an anticancer drug, and the mechanism of CPX in treating colorectal cancer has also been elucidated: the downregulation of DJ-1 is the key mechanism for the anticancer activity of CPX (52). Perinatal exposure to diethylstilbestrol (DES) increases the risk of all cancers by at least twofold (53). DES is used as an antiprostate tumor drug by promoting cell cycle arrest and apoptosis of prostate cancer cells (54). Furthermore, studies have shown that diethylstilbestrol, an endocrine disruptor, was once used to treat gynecological problems, but it was later confirmed to be carcinogenic (55); this might be explained by the results of diethylstilbestrol activating PDIA3, which is a poor prognostic factor of many cancers. However, diethylstilbestrol is widely used in the treatment of prostate cancer (56); this also can be explained by the fact that PDIA3 is a protective factor for PRAD. Although the potential molecular mechanism of diethylstilbestrol for the treatment of prostate cancer has not yet been elucidated, our results and derived assumptions might provide some clues for further investigations.

How to target the PDIA3 is a constructive topic for cancer therapy. As usual, antibodies and inhibitors are the main targeting methods, but each way of targeting PDIA3 has its own merits. Using antibody to target PDIA3 will be more precise, but antibody design and production are more expensive. It is also reported that the expression of PDIA3 autoantibody increased the risk of miscarriage in thyroid women with thyroid autoimmunity (57); this suggested that PDIA3 antibody may result in associated adverse complications. Several small compounds have been identified as PDIA3 inhibitors, like punicalagin (58), 16F16 (59), and LOC14 (60), and all of them can inhibit the function of PDIA3 by binding it. But only punicalagin is thought to be a specific inhibitor of PDIA3, and 16F16 is a pan-PDI inhibitor; as for LOC14, it is a reversible PDI inhibitor that binds adjacent to the CGHC active site (61). Thus, choosing the suitable inhibitor of PDIA3 is needed to target PDIA3 for cancer therapy.

Admittedly, our research has several limitations. Although we determined that PDIA3 associated with a potential drug by CMap analysis, we could not demonstrate the direct interactions between PDIA3 and these components, and the underlying mechanisms are also still unknown. Furthermore, our study collected sequencing data from open databases for analysis, which inevitably has systematic bias; we believe that well-designed experiments are needed for clinical application and mechanism investigations.

In conclusion, a comprehensive pan-cancer analysis of PDIA3 was conducted, indicating its potential function as a prognostic biomarker for cancers and its potential function of effectively predicting immunotherapy response. A high expression of PDIA3 correlates with prognosis, immune regulators, immune cell infiltration, tumor microenvironment, TMB, and MSI in various cancers; hence, we believe that PDIA3 blocking may be an efficient and feasible therapy for cancers.

## Data Availability Statement

Publicly available datasets were analyzed in this study. These data can be found here: https://xenabrowser.net/datapages.

## Ethics Statement

The studies involving human participants were reviewed and approved by the Ethics Committee of The Second Affiliated Hospital of Nanchang University. The patients/participants provided their written informed consent to participate in this study.

## Author Contributions

KH, XZ, and JL designed this study and revised the manuscript. ZT and QO conducted the data collection, bioinformatic and statistical analysis, figure visualization, and manuscript writing. XL did the revision of this study. All coauthors have approved the final version of the manuscript.

## Funding

The research project is supported by the National Natural Science Foundation (grant nos. 81860448, 82002660, and 82172989), the Natural Science Foundation of Jiangxi Province (grant nos. 20192BAB205077 and 20202ACB216004), the Jiangxi Key Research and Development Projects—Key Project (20212BBG71012), Jiangxi Provincial Science and Technology Innovation Base Plan—Provincial Key Laboratory (20212BCD42008), Construction of Science and Technology Innovation Base—Clinical Medicine Research Center (2021ZDG02001), Jiangxi Key Research and development projects (20212BBG73021), and Province-Youth Talent Project (20212BCJ23023).

## Conflict of Interest

The authors declare that the research was conducted in the absence of any commercial or financial relationships that could be construed as a potential conflict of interest.

## Publisher’s Note

All claims expressed in this article are solely those of the authors and do not necessarily represent those of their affiliated organizations, or those of the publisher, the editors and the reviewers. Any product that may be evaluated in this article, or claim that may be made by its manufacturer, is not guaranteed or endorsed by the publisher.

## Supplementary Material

Supplementary Figure 1
**(A)** The expression levels of PDIA3 in 21 types of cancer cell lines in the CCLE dataset. **(B)** The expression levels of 31 types of tissues in the GTEx datasets.

Supplementary Figure 2
**(A)** The protein expression levels of PDIA3 in LN229 and SW1088 cells after transfected with sh-Control and three sh-PDIA3 plasmids. **(B, C)** The colony formation assay indicated that knockdown of PDIA3 results the decreased colony formation ability of LN229 and SW1088 cells. **(D, E)** The transwell invasion assay indicated that knockdown of PDIA3 weakened the invasive ability of LN229 and SW1088 cells.
