# Supplementary figures and images for: Protein Disulfide-Isomerase A3 Is a Robust Prognostic Biomarker for Cancers and Predicts the Immunotherapy Response Effectively
Source: Front Immunol. 2022 Mar 25;13:837512. doi: 10.3389/fimmu.2022.837512 (PMC8989738; doi:10.3389/fimmu.2022.837512)

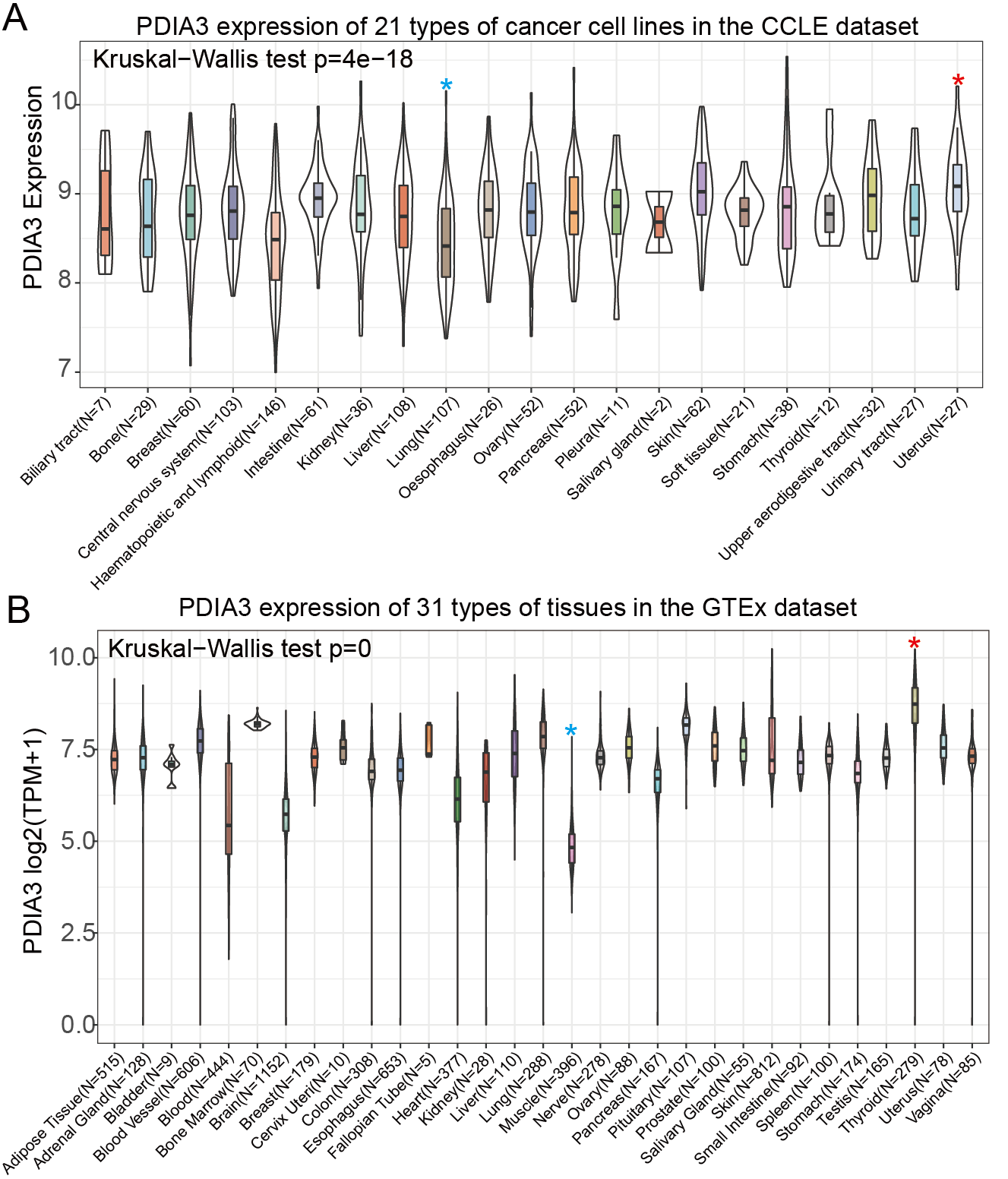

Supplement: Supplementary Figure 1 — (A) The expression levels of PDIA3 in 21 types of cancer cell lines in the CCLE dataset. (B) The expression levels of 31 types of tissues in the GTEx datasets. [file Image_1.tif]

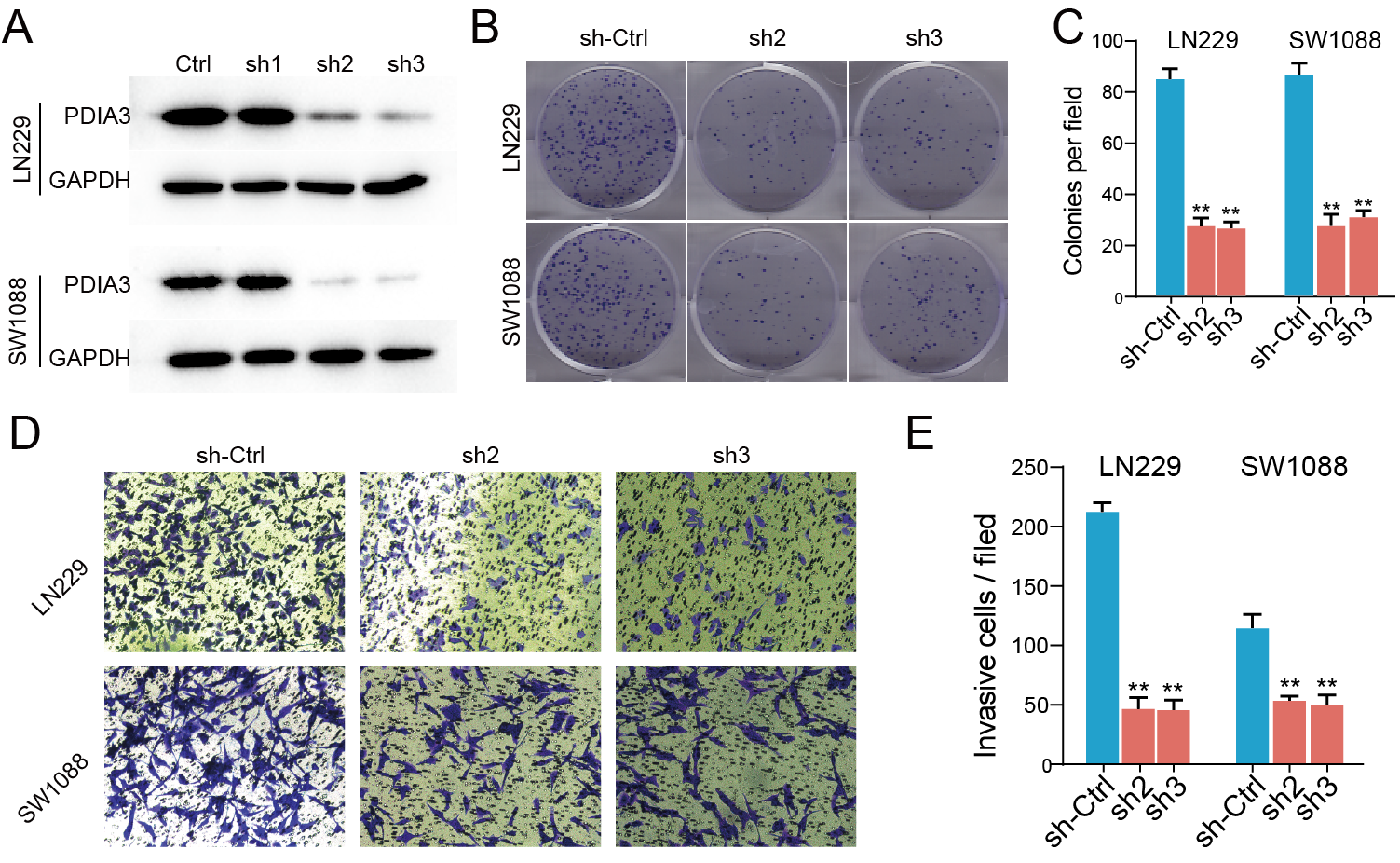

Supplement: Supplementary Figure 2 — (A) The protein expression levels of PDIA3 in LN229 and SW1088 cells after transfected with sh-Control and three sh-PDIA3 plasmids. (B, C) The colony formation assay indicated that knockdown of PDIA3 results the decreased colony formation ability of LN229 and SW1088 cells. (D, E) The transwell invasion assay indicated that knockdown of PDIA3 weakened the invasive ability of LN229 and SW1088 cells. [file Image_2.tif]
